# A Novel CTC-Binding Probe: Enzymatic vs. Shear Stress-Based Detachment Approaches

**DOI:** 10.3390/diagnostics15151876

**Published:** 2025-07-26

**Authors:** Sophia Krakowski, Sara Campos, Henri Wolff, Gabi Bondzio, Felix Hehnen, Michael Lommel, Ulrich Kertzscher, Paul Friedrich Geus

**Affiliations:** 1 Deutsches Herzzentrum der Charité, Institute of Computer-Assisted Cardiovascular Medicine, Augustenburger Platz 1, 13353 Berlin, Germany; henri.wolff@dhzc-charite.de (H.W.); felix.hehnen@dhzc-charite.de (F.H.); michael.lommel@charite.de (M.L.); ulrich.kertzscher@dhzc-charite.de (U.K.); paul-friedrich.geus@charite.de (P.F.G.); 2 Charité—Universitätsmedizin Berlin, Corporate Member of Freie Universität Berlin and Humboldt-Universität zu Berlin, Charitéplatz 1, 10117 Berlin, Germany; 3 Invicol GmbH, Köpenicker Str. 325, 12555 Berlin, Germany; s.campos@invicol.com (S.C.); g.bondzio@invicol.com (G.B.)

**Keywords:** circulating tumor cells (CTC), TrypLE™, BMProbe™, cell detachment, wall shear stress

## Abstract

**Background/Objectives**: Liquid biopsy is a minimally invasive alternative to tissue biopsy and is used to obtain information about a disease from a blood sample or other body fluids. In the context of cancer, circulating tumor cells (CTC) can be used as biomarkers to determine the nature of the tumor, its stage of progression, and the efficiency of the administered therapy through monitoring. However, the low concentration of CTCs in blood (1–10 cells/mL) is a challenge for their isolation. Therefore, a minimally invasive medical device (BMProbe™) was developed that isolates CTCs via antigen–antibody binding directly from the bloodstream. Current investigations focus on the process of detaching bound cells from the BMProbe™ surface for cell cultivation and subsequent drug testing to enable personalized therapy planning. **Methods**: This article presents two approaches for detaching LNCaP cells from anti-EpCAM coated BMProbes™: enzymatic detachment using TrypLE™ and detachment through enzymatic pretreatment with supplementary flow-induced shear stress. The additional shear stress is intended to increase the detachment efficiency. To determine the flow rate required to gently detach the cells, a computational fluid dynamics (CFD) simulation was carried out. **Results**: The experimental test results demonstrate that 91% of the bound cells can be detached enzymatically within 10 min. Based on the simulation, a maximum flow rate of 47.76 mL/min was defined in the flow detachment system, causing an average shear stress of 8.4 Pa at the probe edges. The additional flow treatment did not increase the CTC detachment efficiency. **Conclusions**: It is feasible that the detachment efficiency can be further increased by a longer enzymatic incubation time or higher shear stress. The influence on the integrity and viability of cells must, however, be considered.

## 1. Introduction

Cancer diseases are the reason for almost one in six deaths (16.8%) worldwide. Demographic forecasts predict that the number of new cancer cases will rise to 35 million per year by 2050 [1]. Diagnostic delays can have a significant impact on patient outcomes, and the financial burden of cancer treatment can increase considerably as the disease progresses. Early detection of the disease and efficient therapy are therefore of crucial importance [2].

The standard of tumor diagnosis includes various imaging techniques and a solid biopsy to determine tumor histology and staging. However, the procedure to collect tissue biopsies involves limitations and risks such as infections or bleeding [2,3,4]. Furthermore, it merely reflects the tumor at the time of sampling and is therefore only suitable for progress monitoring to a limited extent [5]. Also, a tumor mass is not an isolated entity. It consists of different cells that communicate with the surrounding microenvironment. This environment influences their growth, progression, and response to treatment [6]. A tissue sample can therefore only represent a fraction of the tumor mass, and if the sample obtained is too small, this can result in misdiagnosis [2]. Moreover, in some tumor diseases, biopsy sampling is restricted, as certain regions are difficult or impossible to access for tissue sampling.

In this context liquid biopsy is becoming increasingly important to improve diagnosis and therapy monitoring as a less stressful alternative to invasive tissue biopsy. Liquid biopsy focuses on the isolation and analysis of biomarkers like circulating tumor cells (CTCs), cell-free DNA and RNA and exosomes from the blood or other body fluids such as urine, saliva and cerebrospinal fluid [7,8].

**Figure 1:**
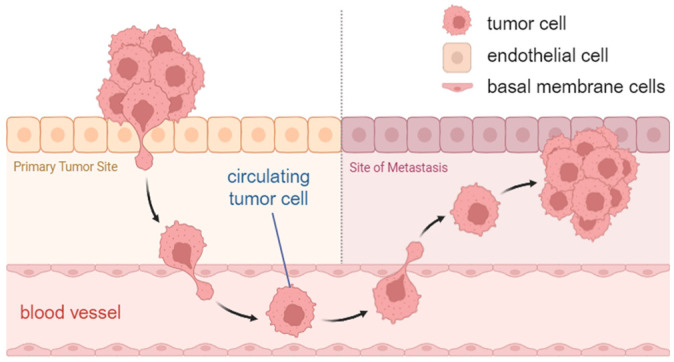
Formation of metastasis: release of cells from the primary tumor, entrance into the bloodstream, and infiltration from the blood vessels into distant tissues. Created with BioRender.com.

CTCs are cells that have detached from tumor sites and subsequently circulate through the bloodstream. They may cause the formation of metastases (Figure 1) and are considered to reflect the genetic heterogeneity of the tumor tissue [9]. Their analysis can reveal clinically relevant information on the patient’s metastatic potential [10,11,12]. CTCs can also be used for diagnosis of cancer and early detection, monitoring therapeutic interventions, and prognostic evaluation, as the detectable number of CTCs increases as the disease progresses [13,14]. Moreover, it is feasible to cultivate isolated CTCs, which enables the investigation of drug efficiency and resistance development for a particular patient. A study from Kapeleris et al. demonstrates that a short-term cell culture can already be established from five tumor cells per seven milliliters of patient blood [15]. Generally, the probability of successful cultivation increases with the count of isolated CTCs [16]. However, in previous studies, the low CTC numbers in blood (1–10 CTCs/mL) and their heterogeneity have been identified as significant challenges in isolation, which currently limits the clinical applications [17,18]. Many liquid biopsy strategies being developed for early cancer detection lack the required sensitivity [2].

**Figure 2:**
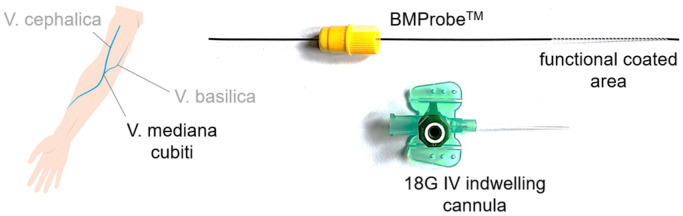
BMProbe™ inserted into a peripheral vein via an indwelling cannula.

To overcome these challenges, a novel medical in vivo device was developed. The BMProbe™ is minimally invasive and isolates CTCs during a 30-min incubation time in the peripheral arm vein due to a specific antibody coating (Figure 2), which can be adapted to different entities. The geometry of the BMProbe™ features 32 windings and has been designed to enhance blood–surface interaction [19]. Thus, it is assumed that a significantly larger proportion of blood can be screened for CTCs than with conventional ex vivo isolation methods [20,21]. A venous flow rate of 2 mL/min results in a yield potential of about 60–600 CTCs within 30 min based on the previously mentioned CTC concentration. The risks of the application are comparable to the risks associated with the insertion of a peripheral venous catheter or routine blood sampling performed in clinical practice. These include immune reactions, blood loss, and mechanical irritation of the vein. However, this risk can be considered minimal due to the elastic material properties and the absence of sharp edges on the BMProbe™. In addition, required tests for biocompatibility (according to ISO 10993 [22,23,24,25,26,27]), chemical (according to ISO 9626 [28]), and mechanical safety (according to ISO 11070 [29]) were carried out to minimize health risks.

A study from Geus et al. shows the functionality of the BMProbe™ for isolating CTCs in ex vivo blood samples [21]. Current investigations focus on the detachment of isolated cells from the probe for subsequent cell cultivation. TrypLE™ is a well-established protease used in cell culture to detach adherent cells from the flask. However, binding strength studies have shown that the binding between the BMProbe™ coating and the target cells is very strong (dissociation constant KD < 1 × 10^−12^ M), which is required for isolation from the bloodstream. Therefore, detachability must be investigated.

This article introduces two CTC detachment approaches. First, the efficiency of enzymatic detachment using TrypLE™ was examined. Subsequently, an attempt was made to increase the detachment efficiency by applying an additional flow-induced shear stress using a flow detachment system. A computational fluid dynamics (CFD) simulation was performed to estimate the required flow rate. It should be noted that the subsequent cultivation of detached cells is not covered by this study.

## 2. Materials and Methods

### 2.1. CFD Simulation

Blood flowing through the body’s vessels causes shear stress on the walls. In the simple case of a two-dimensional Couette flow, wall shear stress (τ) is calculated using the velocity (du) normal to the wall, the distance between the wall and the flow layer dy, and the fluid’s dynamic viscosity (µ): τ = µ‧du/dy [30]. This mechanical stress influences the morphology, the physiological behavior, and the adhesion properties of cells [31]. In the human vascular system, the wall shear stress (WSS) can vary greatly from less than 1 Pa to 10 Pa [30,32,33]. It is necessary to determine the required flow rate for the intended mechanical detachment of the cells by WSS. To avoid cell damage, the maximum WSS must be applied within the physiological range. For this study, it is specified that the WSS should not exceed a limit of 9 Pa.

**Figure 3:**
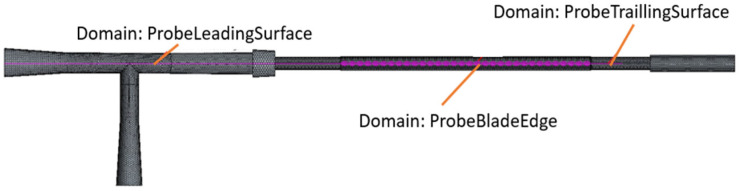
Imported CAD model created with SolidWorks: geometry of the probe inserted into the tube with defined domains in Star-CCM+.

Therefore, a numerical analysis of the flow around the BMProbe™ (Invicol GmbH, Berlin, Germany) was performed. The 3D geometry of the BMProbe™ and the tube of the detachment system was constructed using the computer-aided design (CAD) software SolidWorks (Dassault Systèmes, Vélizy-Villacoublay, France, Version 2023 SP 2.1). The geometry was then exported to the CFD software package STAR-CCM+ (Siemens Digital Industries Software, Plano, TX, USA, Version 16.04.007) to perform the simulations (Figure 3). The fluid was modeled as Newtonian with a constant density of 1005 kg/m^3^ [34] and a dynamic viscosity of 1.00 mPa‧s [35], consistent with fluid properties of PBS (Phosphate-Buffered Saline) at 20 °C. The flow was assumed to be laminar based on a Reynolds number of 508 using a channel diameter of 2 mm and the finally selected flow rate. The channel walls and the BMProbe™ surface were set as no-slip boundaries, and a constant pressure was applied at the outlet. A mass flow inlet with a variable mass flow was created to determine the required flow conditions.

The tube geometry was discretized using a finite volume mesh approach by dividing the geometry into polyhedral mesh elements. The in- and outlet were extended by 20 mm to ensure solution independence from the prescribed boundary conditions using a conformal interface with prismatic cell elements. To better capture the high velocity gradients forming in the near-wall region of the BMProbe™, five layers of prismatic elements were extruded from the surface mesh, forming a 50 µm thick near-wall prism layer. The “Segregated Flow” solver was used to sequentially solve the mass and momentum equations with the SIMPLE algorithm (STAR-CCM+, Version 16.04.007, User Guide/Segregated Flow Model Family Reference) with a second-order upwind convection scheme.

The mesh dependency of the solution was analyzed by varying the base size parameter of STAR-CCM’s polyhedral mesher and comparing the solution between meshes. Varying the base size changes the average distance between elements and thus influences the overall number of elements in the mesh. Mesh independence was studied for the surface average of the WSS on a representative section of the probe in the middle of the channel. The deviation from the finest mesh shows a convergent behavior when increasing the number of elements. The mesh with a base size of 400 µm, which corresponds to nearly three million elements, was selected for the simulation due to a low deviation of 1.72% from the finest mesh.

### 2.2. Detachment Methods

In this chapter, the two approaches for detaching CTCs from the BMProbe™ are described. Both methods use LNCaP cell line cells (Lymph Node Carcinoma of the Prostate), which were detached from the culture flask, washed, and stained with the DNA-specific dye Hoechst 33342 (Thermo Fisher Scientific, Waltham, MA, USA; 2 nmol, 10 min, 37 °C) to make them visible under a fluorescence microscope. The stained cells were suspended in cell medium (10% Dextran + 10% Fetal Calf Serum + PBS (enzymatic) or RPMI + 10% Fetal Calf Serum + 1% antibiotic/antimycotic solution (enzymatic + flow), Thermo Fisher Scientific, Waltham, MA, USA; 5000 cells/mL). Since EpCAM (Epithelial Cell Adhesion Molecule) antigen is found on prostate cancer cells and other tumor entities, anti-EpCAM (CD326 antibody, Miltenyi Biotec, Bergisch Gladbach, Germany) coated BMProbes™ were used to bind the tumor cells [9,36,37].

**Figure 4:**
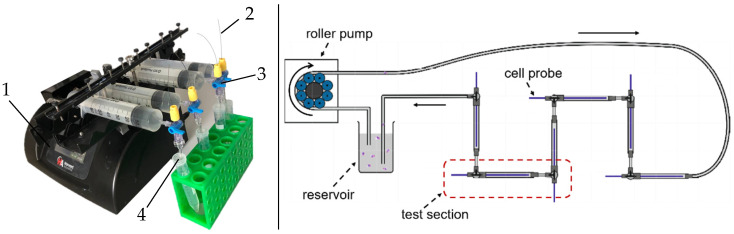
Test Stands; **left**: Setup for flow detachment: (1) syringe pump, (2) BMProbe™, (3) stopcock, (4) tube; **right**: Flow system for attachment of LNCaP cells on the BMProbe™ [38].

For cell attachment to the BMProbe™, a ∅ 2 mm flow system was used, introduced by Hehnen et al. [38] (Figure 4). The flow system was previously treated with a 3% BSA (Bovine Serum Albumin) solution for 30 min at room temperature (RT) to prevent the attachment of proteins and cells to the tube walls.

Afterwards, the system was cleaned with sterile PBS, and four BMProbes™ were inserted into the respective three-way stopcock. The cell suspension consisting of cell medium and stained LNCaP cells was flushed through the system at 3.77 mL/min, which roughly corresponds to the volume flow in the vena mediana cubiti [39,40], and the BMProbes™ were incubated for 30 min at RT. Subsequently, the BMProbes™ were removed from the flow system and washed in sterile PBS for 5 min. Cells bound to the probe surface were counted in a wet chamber filled with PBS + 1% Penicillin-Streptomycin + 1% Amphotericin B under a fluorescence microscope.

#### 2.2.1. Enzymatic Detachment

**Figure 5:**
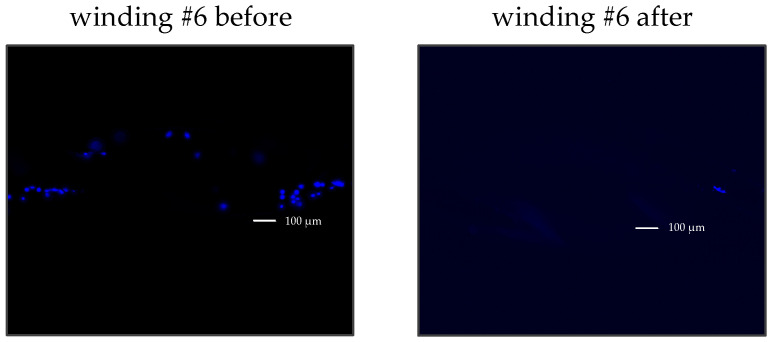
Example: LNCaP cells (blue) stained with Hoechst 33342 bound to winding #6 on a BMProbe™; **left**: before enzymatic detachment, **right**: after enzymatic detachment.

To detach the cells, the BMProbes™ were incubated in prewarmed TrypLE™ (TrypLE Express Enzyme (no additive), Thermo Fisher Scientific, Waltham, MA, USA) at 37 °C with shaking (MTS 2/4 digital microtiter shaker, IKA, Staufen, Germany) for 10 min at 125 rpm. The number of remaining bound cells after treatment was subsequently counted under a fluorescence microscope (Figure 5). The detachment efficiency (DE) was calculated by comparing the cell counts on the BMProbe™ surface before and after the detachment process.

#### 2.2.2. Enzymatic + Flow Detachment

The flow detachment experiment was divided into two steps: pretreatment with the bond-cleaving enzyme TrypLE™ and detachment through flow-induced shear stress. Consideration of flow detachment without enzymatic pretreatment was excluded, as the measured binding strength between the BMProbe™ and the cells (dissociation constant KD < 1 × 10^−12^ M) rules out detachment without destroying the cells.

For the enzymatic pretreatment of the cells, the BMProbes™ were inserted into tubes containing prewarmed TrypLE™ and incubated at 37 °C for 10 min, analogous to the enzymatic method, but without shaking.

Afterwards, the tube-probe units were connected to the flow detachment system consisting of a syringe pump (Harvard PHD ULTRA™ Syringe Pump 70-3007, Holliston, MA, USA), PBS-filled syringes, stopcocks, and tubes (Figure 4). The pump generated a linear increasing flow rate of 0.01–47.76 mL/min (see Section 3.1) in 6 s to detach the cells. The increase is designed to guarantee that each cell is exposed to the minimum amount of stress required. Hence, no cell damage is expected due to the method. The DE was calculated as described in Section 2.2.1 (Figure 5).

## 3. Results

### 3.1. Results of CFD Simulation

**Figure 6:**
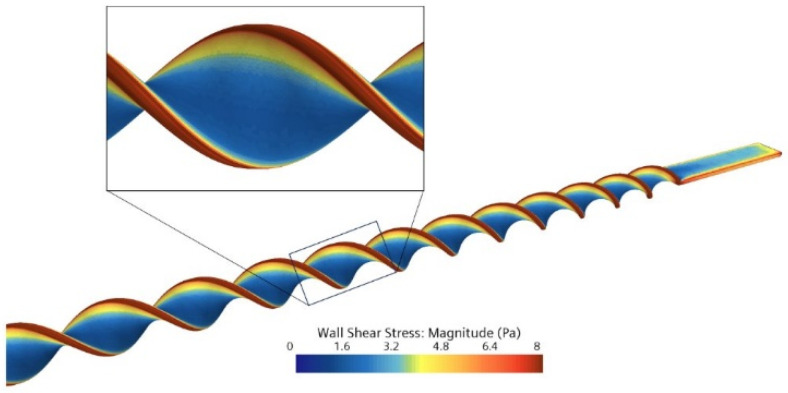
CFD simulation of wall shear stress on the probe surface using a volume flow of 47.76 mL/min.

The numerical simulations were conducted in accordance with the required specifications for cell-friendly detachment from the BMProbe™. The flow rate of 47.76 mL/min generates an average WSS of 8.4 Pa at the edges, where the highest WSS values are observed (Figure 6). This corresponds approximately to the maximum physiological WSS in the human body. Therefore, this flow rate is defined as maximum for investigating detachment by flow.

### 3.2. Combined Results of Both Detachment Methods

**Figure 7:**
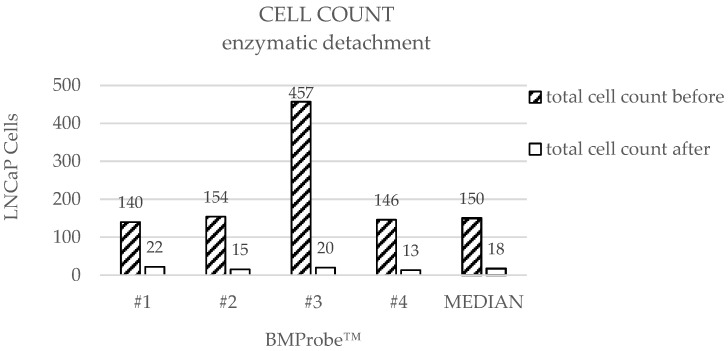
Cell counts on the BMProbe™ with conjugated anti-EpCAM before and after treatment. Cell yield: #1: 118, #2: 139, #3: 437, #4: 133, median cell yield: 132.

**Figure 8:**
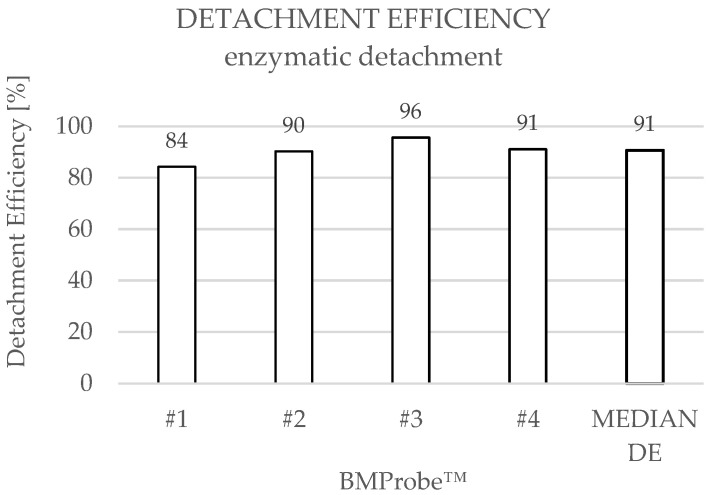
Detachment efficiencies (DE) when using enzymatic treatment.

LNCaP cells were detached from BMProbes™ using two different methods. In the case of enzymatic detachment, the median cell count before detachment was 150 (range: 140–457), while the median cell count after detachment was 18 (range: 13–22) (Figure 7). This results in a cell yield between 118 and 437 cells and a median detachment efficiency (DE) of 91% (Figure 8). An increased number of bound cells can be found on probe #3 before detachment compared to the other BMProbes™. Ignoring probe #3, the cell yield is between 118 and 133 cells, and the mean DE is 89% with a standard deviation (SD) of 3.03%.

**Figure 9:**
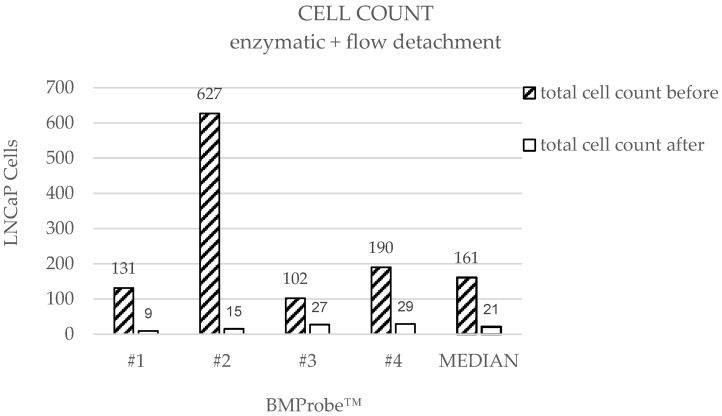
Cell counts on the BMProbe™ with conjugated anti-EpCAM before and after treatment. Cell yield: #1: 122, #2: 612, #3: 75, #4: 161, median cell yield: 140.

**Figure 10:**
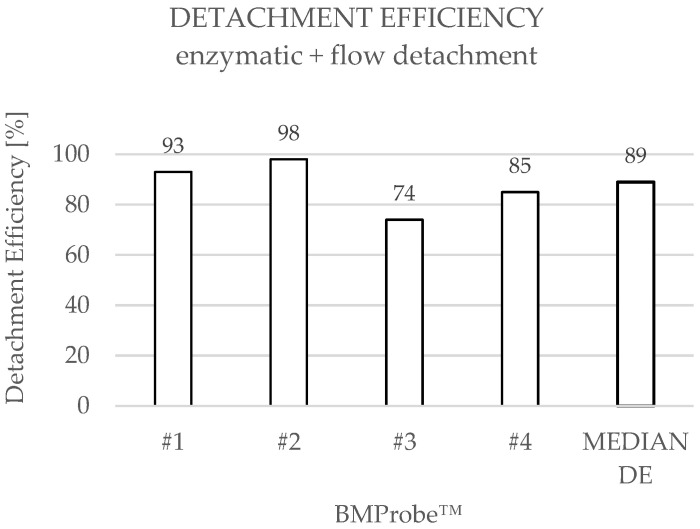
Detachment efficiencies (DE) when using enzymatic treatment and a flow rate of 47.76 mL/min in the flow detachment system.

The results of the detachment by flow-induced shear stresses demonstrate that the median of cell count is 161 (range: 102–627) before and 21 (range: 9–29) after detachment (Figure 9) with a cell yield between 75 and 612 cells. The median DE is 89% (Figure 10). Probe #2 shows an increased number of bound cells prior to detachment compared to the other BMProbes™. Ignoring Probe #2, the cell yield is between 75 and 161 cells, and the mean DE is 84% with a standard deviation (SD) of 7.97%.

The median cell counts and the median DEs show similar results for both approaches. A comparison of the mean DEs, excluding outliers, indicates a slightly higher value for enzymatic detachment, which is negligible because of the small sample size and the SD.

## 4. Discussion

This article presents two suitable approaches to detach CTCs from a minimally invasive cell-binding probe.

However, the complex shape of the BMProbe™ presents a challenge regarding the assessment of the flow field in the surrounding area via CFD. Nevertheless, the tube geometry is relatively simple and the flow velocities are low, thus allowing for the assumption that the numerical simulation of the flow around the BMProbe™ is sufficiently accurate [38].

It should also be noted that the simulation setup was designed with a BMProbe™ situated in a central position within the tube for a precise statistical assessment of experiments with fewer tests. In practical use, the BMProbe™ bends over its length and thus has an angle that creates variable flow conditions during the detachment process. Therefore, the exact WSS at the BMProbe™ may deviate from the simulation, and the determined flow rate can only be transferred to the experimental setup to a limited extent.

An examination of the experimental results obtained from both detachment methods reveals comparable detachment efficiencies. However, additional treatment of the cells with flow-induced shear stresses did not increase the detachment efficiency compared to enzymatic detachment. Nevertheless, both methods are suitable for CTC detachment since the median detachment efficiency is 91% for enzymatic detachment and 89% for additional flow detachment.

As expected, the median cell counts before detachment are also similar, since cell attachment was achieved using the same method in both approaches. A review of the cell counts before detachment for each individual BMProbe™ reveals a considerably higher cell count at probe #3 (Figure 7) for enzymatic detachment and probe #2 for flow detachment (Figure 9). The DE may therefore be overestimated. This phenomenon is potentially the result of deviations in the coating process. However, excluding the outliers from the analysis, the mean DE for enzymatic detachment is 89% and for flow detachment 84%, which still confirms the suitability of the methods.

The cell counts can also be influenced by the flow system used for attachment. According to Geus et al., cells can be potentially damaged by non-physiological characteristics created by stopcocks [21].

When comparing the two detachment methods, different media were used for the LNCaP cells, but no influence on the cell properties is expected, as both media are well-established and commonly used.

Looking at the number of cells detached from the BMProbes™, they all exceed 70 cells, which is sufficient to set up a 2D cell culture according to the literature [15]. It should be noted, though, that the CTC concentration used for cell attachment on the BMProbes™ was over 500× higher compared to patient blood to achieve an accurate assessment of detachment efficiency and to consider the cell loss within the flow system [21]. The cell counts, therefore, cannot be transferred to the in vivo application. Nevertheless, the in vivo yield potential of up to 600 cells within 30 min using the BMProbe™ is promising regarding a sufficient cell count for cultivation.

To enhance the reliability of the experimental outcomes, additional investigations are necessary. By increasing the enzymatic incubation time or the flow rate, it is also feasible to improve the detachment efficiency. It is important to note that the subsequent cultivation of cells requires their integrity. The cell viability after the detachment process, e.g., using trypan blue to mark dead cells, and possible changes in cell properties due to enzymatic treatment, must be analyzed. Therefore, an isolation method for the detached cells must also be established. Further, additional studies should be conducted with realistic cell numbers to more accurately predict the efficiency of the methods for an in vivo application. Finally, the cultivation of detached cells needs to be established and carried out as the next step towards resistance prediction and personalized therapy.

## Data Availability

The original contributions presented in this study are included in the article. Further inquiries can be directed to the corresponding author.

## Abbreviations

The following abbreviations are used in this manuscript:

CAD	Computer-Aided Design
CFD	Computational Fluid Dynamics
CTC	Circulating Tumor Cell
DE	Detachment Efficiency
EpCAM	Epithelial Cell Adhesion Molecule
LNCaP	Lymph Node Carcinoma of the Prostate
SD	Standard Deviation
WSS	Wall Shear Stress
